# Anti-ganglionic acetylcholine receptor antibodies in functional neurological symptom disorder/conversion disorder

**DOI:** 10.3389/fneur.2023.1137958

**Published:** 2023-02-13

**Authors:** Ryusei Nagata, Eiji Matsuura, Satoshi Nozuma, Mika Dozono, Yutaka Noguchi, Masahiro Ando, Yu Hiramatsu, Daisuke Kodama, Masakazu Tanaka, Ryuji Kubota, Munekazu Yamakuchi, Yujiro Higuchi, Yusuke Sakiyama, Hitoshi Arata, Keiko Higashi, Teruto Hashiguchi, Shunya Nakane, Hiroshi Takashima

**Affiliations:** ^1^Department of Neurology and Geriatrics, Kagoshima University Graduate School of Medical and Dental Sciences, Kagoshima, Japan; ^2^Division of Neuroimmunology, Joint Research Center for Human Retrovirus Infection, Kagoshima University, Kagoshima, Japan; ^3^Department of Laboratory and Vascular Medicine, Kagoshima University Graduate School of Medical and Dental Sciences, Kagoshima, Japan; ^4^Department of Neurology, Graduate School of Medicine, Nippon Medical School, Tokyo, Japan

**Keywords:** autonomic symptoms, functional neurological symptom disorder, conversion disorder, ganglionic acetylcholine receptor, antibody, LIPS

## Abstract

**Objective:**

Autoimmune autonomic ganglionopathy (AAG) is a rare disorder characterized by autonomic failure associated with the presence of anti-ganglionic acetylcholine receptor (gAChR) antibodies; however, several studies have reported that individuals with anti-gAChR antibodies present with central nervous system (CNS) symptoms such as impaired consciousness and seizures. In the present study, we investigated whether the presence of serum anti-gAChR antibodies correlated with autonomic symptoms in patients with functional neurological symptom disorder/conversion disorder (FNSD/CD).

**Methods:**

Clinical data were collected for 59 patients presenting with neurologically unexplained motor and sensory symptoms at the Department of Neurology and Geriatrics between January 2013 and October 2017 and who were ultimately diagnosed with FNSD/CD according to the Diagnostic and Statistical Manual of Mental Disorders, 5th Edition. Correlations between serum anti-gAChR antibodies and clinical symptoms and laboratory data were analyzed. Data analysis was conducted in 2021.

**Results:**

Of the 59 patients with FNSD/CD, 52 (88.1%) exhibited autonomic disturbances and 16 (27.1%) were positive for serum anti-gAChR antibodies. Cardiovascular autonomic dysfunction, including orthostatic hypotension, was significantly more prevalent (75.0 vs. 34.9%, *P* = 0.008), whereas involuntary movements were significantly less prevalent (31.3 vs. 69.8%, *P* = 0.007), among anti-gAChR antibody-positive compared with -negative patients. Anti-gAChR antibody serostatus did not correlate significantly with the frequency of other autonomic, sensory, or motor symptoms analyzed.

**Conclusions:**

An autoimmune mechanism mediated by anti-gAChR antibodies may be involved in disease etiology in a subgroup of FNSD/CD patients.

## 1. Introduction

Functional neurological symptom disorder/conversion disorder (FNSD/CD) is a neurological disorder of voluntary movement or sensory function with no medial or neurological cause; and is described in the Diagnostic and Statistical Manual of Mental Disorders, Fifth Edition (DSM-5); as a long-term impairment that significantly impairs quality of life and requires assistive and supportive services (1). Because the etiopathogenesis of FNSD/CD is unknown and abnormalities in clinical tests are rare, FNSD/CD is generally considered to be a psychological disorder (2, 3). Diagnosis of FNSD/CD in patients with non-specific motor-sensory symptoms can be difficult, and some are ultimately determined to have tumor-associated neurological syndrome or autoimmune encephalitis (3–6). In addition, elevated blood inflammatory markers have been reported in some FNSD/CD patients (7, 8), leading to the hypothesis that FNSD/CD symptoms may correlate with immune dysfunction. To avoid misdiagnosis, physicians are recommended to not assign a diagnosis of psychogenic reactions to neurological symptoms that are difficult to explain, such as dystonia or atypical seizures. Indeed, DSM-5 and International Classification of Diseases, 11th Revision (ICD-11) no longer supports the term psychogenic factors as a cause of symptoms of neurological disorders such as involuntary movement.

Although patients with FNSD/CD often exhibit autonomic symptoms such as orthostatic hypotension and gastrointestinal dysfunction and dysuria, the exact frequency of autonomic complications in patients with FNSD/CD is unknown (9, 10). The autonomic nervous system is broadly divided into sympathetic and parasympathetic components, with the main neurotransmitters being norepinephrine and acetylcholine (ACh). The two ACh receptor (AChR) subtypes, nicotinic and muscarinic, are widely expressed throughout the body. In both the sympathetic and parasympathetic ganglia, preganglionic to postganglionic fiber neurotransmission is predominantly mediated by ACh and the major receptor involved is the nicotinic AChR (nAChR) (11). nAChRs exist as homodimeric or heteromeric complexes of nine subunits (α2–7 and β2–4) in the mammalian peripheral and central nervous systems (12). The distribution of gAChR in organs other than the nervous system is not clear. In humans, autonomic ganglia nAChRs generally exist as pentameric heterocomplexes, with α3 and β4 subunits playing important roles (13, 14). Autoantibodies against nAChRs have been associated with several diseases, including myasthenia gravis, in which the antibodies target the α1 subunit of nAChRs at the neuromuscular junction (15). Serum antibodies against the autonomic ganglion nAChR (anti-gAChR) were first detected in patients with autonomic dysfunction in 1998 and resulted in the concept of autoimmune autonomic ganglionopathy (AAG), a term that refers to autonomic neuropathy mediated by an autoimmune mechanism (16).

In the present study, we retrospectively reviewed clinicopathological data, including autonomic, motor, and sensory symptoms and anti-gAChR antibody serostatus, in patients who were admitted to our Neurology Department with neurologically unexplainable motor and sensory symptoms and were ultimately diagnosed with FNSD/CD.

## 2. Materials and methods

### 2.1. Patients

Patients with the main complaint of motor and sensory disorders who were admitted to the Department of Neurology and Geriatrics at Kagoshima University Hospital between January 2013 and October 2017 were included in this study. Of those patients, 68 met the DSM-5 diagnostic criteria for FNSD/CD (1). Medical records were retrospectively reviewed for clinical symptoms and blood and cerebrospinal fluid (CSF) test results. After exclusion of patients with concomitant autoimmune neurological disease including Sjögren's syndrome, myasthenia gravis, systemic lupus erythematosus, chronic inflammatory demyelinating polyneuropathy, a total of 59 patients were included in the final analysis set.

### 2.2. Neurological examinations

All patients who presented with motor and sensory symptoms were evaluated neurologically and no neurologic lesion sites were found. Nine of the patients with sensory symptoms had nerve conduction studies performed because of difficulties in determining the site of the lesion by the neurologist. Nerve conduction studies showed that none of the patients had decreased nerve conduction velocity or sensory nerve action potentials.

Routine neurological examinations included autonomic symptoms, which were categorized into four types: gastrointestinal dysfunction (constipation, diarrhea, early satiety, nausea, vomiting, anorexia, abdominal pain, and feeling abdominally bloated); cardiovascular autonomic dysfunction (orthostatic hypotension, postural orthostatic tachycardia syndrome [POTS], tachycardia, and palpitations); bladder dysfunction; and anhidrosis. The diagnostic criteria of the European Society of Cardiology in 2018 were used for the diagnosis of orthostatic hypotension and POTS. Bladder dysfunction was assessed by the presence or absence of incontinence, frequent urination that interfered with daily activities, and overactive bladder as assessed by OABSS (17, 18). Motor symptoms were categorized into three types: weakness, involuntary movements, and ataxia. Sensory symptoms were categorized as either sensory disturbance (abnormal or decreased sensation) or pain (headache, pain in extremities and trunk).

### 2.3. Detection of anti-gAChR antibodies using a luciferase immunoprecipitation system

Serum antibodies against gAChR α3 and β4 subunits were quantified using a LIPS assay (13, 19), in which specific antibodies are bound to a *Gaussia* luciferase-tagged antigen and the bound antibody is quantified by measuring luciferase activity with a luminometer. The results are expressed as relative luminescence units (RLU). Serum was considered positive for anti-gAChR α3 or β4 antibodies if the RLU of the test sample exceeded a cut-off value of three standard deviations (SDs) greater than the mean RLU of the control sample (sera obtained from healthy individuals). We evaluated the antibody levels as an antibody index (A.I.). A.I.= [measurement value of the sample serum (RLU)]/[the cut-off value (RLU)].

### 2.4. Statistical analysis

Mean values were compared between anti-gAChR antibody-positive and -negative patient groups. Age and laboratory findings were compared using *t*-tests, and gender, clinical symptoms, and other autoantibodies were compared using Fisher's exact test. IBM SPSS, version 27, was used for statistical analyses. *P* < 0.05 was considered significant.

## 3. Results

**Figure 1:**
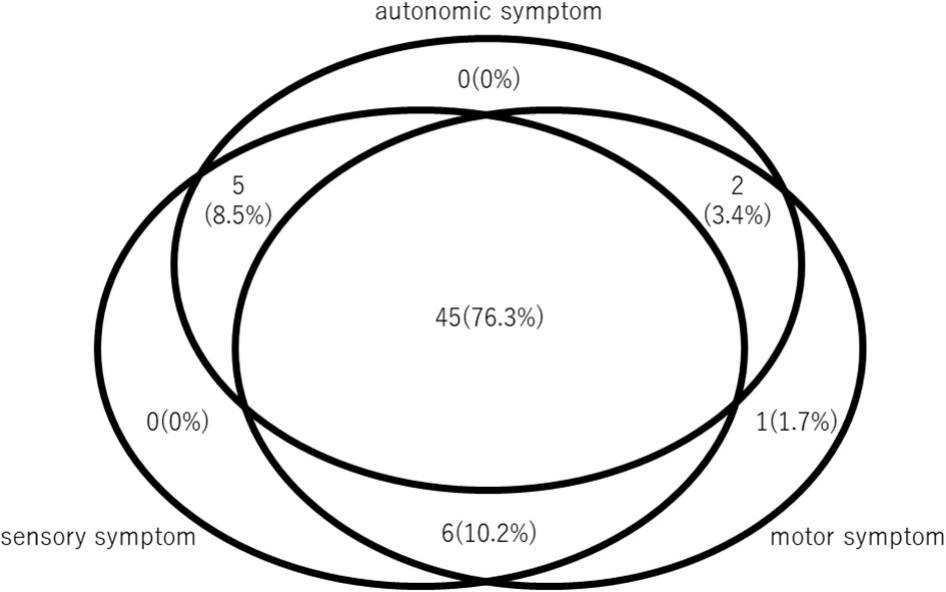
Prevalence of symptoms in 59 patients with FNSD/CD. Venn diagram shows the number and percentage of patients with autonomic, sensory, and motor symptoms.

Of the 68 patients who presented with motor or sensory deficits and met the diagnostic criteria for FNSD/CD, 9 had concomitant neuroimmune disease and were excluded. The final analysis set consisted of 59 patients (Table 1), of whom 51 (86.4%) were female. The mean (±SD) age at admission was 26.4 ± 15.4 years, the age at symptom onset was 22.0 ± 14.9 years, and the duration of illness was 4.4 ± 5.9 years. We examined the prevalence of motor, sensory, and autonomic symptoms, and found that 45 of the 59 patients (76.3%) had all three symptom types, one (1.7%) had only motor symptoms, and none had only sensory symptoms (Figure 1). Of the 59 patients, 52 (88.1%) had autonomic symptoms that included gastrointestinal symptoms (40 patients, 67.8%), cardiovascular autonomic symptoms (27 patients, 45.8%), bladder dysfunction (23 patients, 39.0%), and anhidrosis (16 patients, 27.1%) (Table 1).

**Table 1:** Characteristics of patients with FNSD/CD (*N* = 59).

Male		**8 (13.6%)**
Age at admission (y.o.)		26.4 ± 15.4
Age at onset (y.o.)		22.0 ± 14.9
Disease duration (year)		4.4 ± 5.9
**Autonomic dysfunction**		52 (88.1%)
	Gastrointestinal dysfunction	40 (67.8%)
	Cardiovascular autonomic dysfunction	27 (45.8%)
	Bladder dysfunction	23 (39.0%)
	Anhidrosis	16 (27.1%)
**Sensory symptom**		56 (95.0%)
	Pain	52 (88.1%)
	Sensory disturbance	43 (72.9%)
**Motor symptom**		54 (91.5%)
	Weakness	47 (79.7%)
	Involuntary movement	35 (59.3%)
	Ataxia	9 (15.3%)

**Figure 2:**
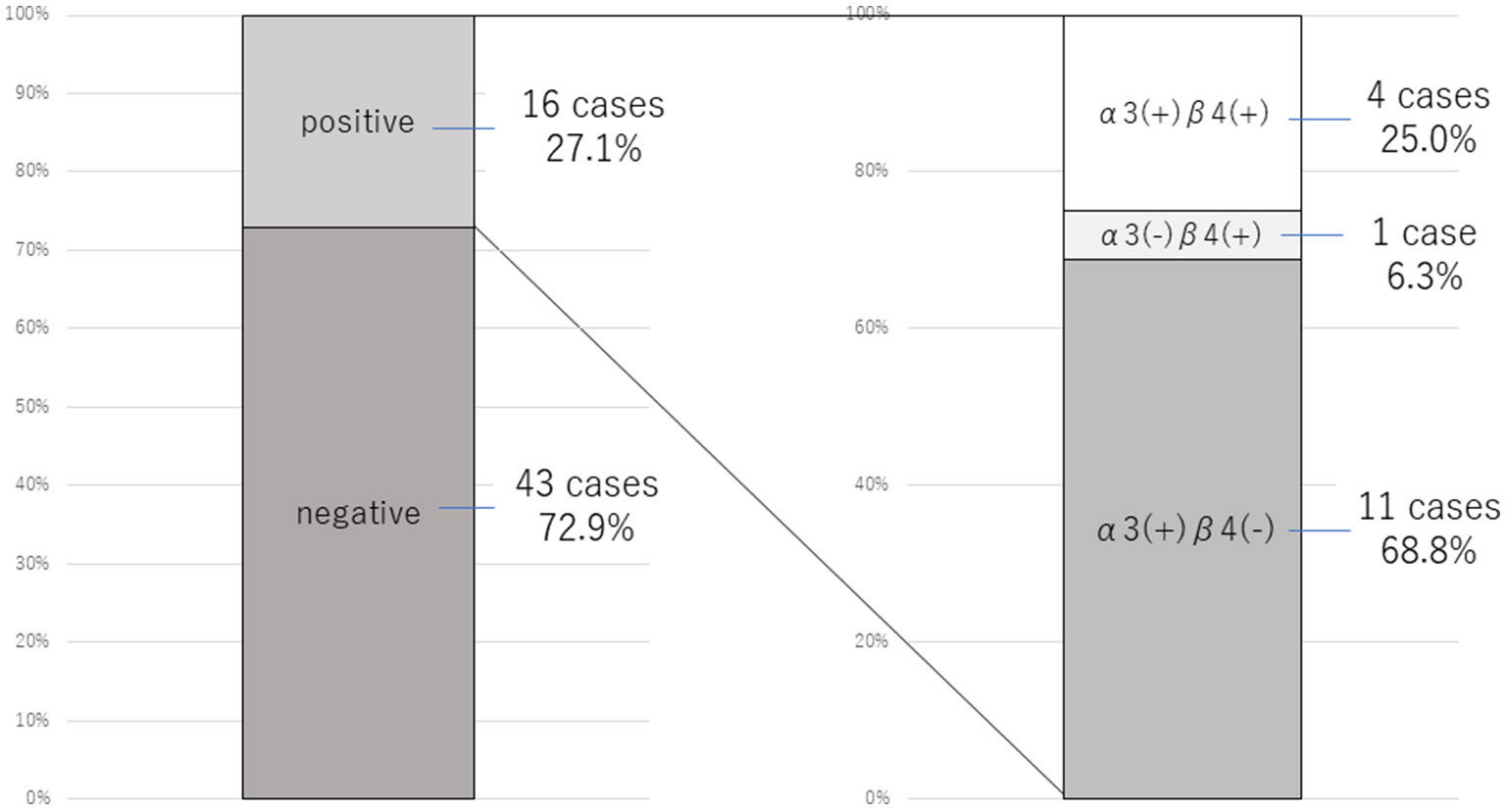
Prevalence of anti-gAChR antibodies in 59 patients with FNSD/CD. The proportion of patients positive for anti-gAChR antibodies (left) and for antibodies specifically against the α3 and β4 subunits (right) are shown.

Because of the prevalence of autonomic symptoms among our patient cohort, we investigated the possible relationship between FNSD/CD and anti-gAChR antibodies. Of the total cohort of 59 patients, 16 (27.1%) were positive for anti-gAChR antibodies, and of these, 11 were positive for antibodies against the α3 subunit only, one patient was positive for antibodies against the β4 subunit only, and four patients were positive for antibodies against both the α3 and β4 subunits (Figure 2).

We next compared clinical symptoms between patients who were positive (*n* = 16) and negative (*n* = 43) for anti-gAChR antibodies (Table 2). There were no significant differences between the antibody-positive and -negative groups with respect to sex ratio (3 males [18.8%] and 5 males [11.6%], respectively), mean age at admission (30.7 ± 17.8 years and 24.8 ± 14.5 years, respectively), or mean age at disease onset (23.3 ± 16.8 years and 21.5 ± 14.4 years, respectively). There was no significant difference in the prevalence of autonomic dysfunction between the anti-gAChR antibody-positive group (15 of 16 patients, 93.8%) and the antibody-negative group (37 of 43 patients, 86.0%); however cardiovascular autonomic symptoms were significantly more common in the antibody-positive group (75.0 vs. 34.9%, *P* = 0.008). Among the individual cardiovascular autonomic symptoms, POTS and orthostatic hypotension were both more commonly seen among the antibody-positive group, but only the frequency of POTS reached the level of statistical significance (50.0 vs. 9.3%, *P* = 0.002). The prevalence of POTS is 0.5–1.0% of the population, mostly female (female/male: 5:1), so the prevalence in our patients is high regardless of the presence of antibodies. There were no significant association between anti-gAChR antibody serostatus and other autonomic dysfunctions. Although most motor and sensory symptoms were less common in the anti-gAChR antibody-positive group than the antibody-negative group, only the frequency of patients with involuntary movements was significantly different between the two groups (31.3 vs. 69.8%, *P* = 0.007).

**Table 2:** Characteristics of FNSD/CD patients according to anti-gAChR antibody serostatus.

			**Anti-gAChR antibody**		
			**Positive (*****N** =* **16)**	**Negative (*****N** =* **43)**	***P*** **value**
Male			3 (18.8%)	5 (11.6%)	0.670
Age at admission (y.o.)			30.7 ± 17.8	24.8 ± 14.5	0.192
Age at onset (y.o.)			23.3 ± 16.8	21.5 ± 14.4	0.699
Disease duration (year)			7.5 ± 8.8	3.2 ± 4.0	0.079
Anti-gAChR α3 antibodies (A.I.)			1.71 ± 0.59	0.49 ± 0.23	
Anti-gAChR β4 antibodies (A.I.)			0.93 ± 0.46	0.38 ± 0.18	
**Autonomic dysfunction**			15 (93.8%)	37 (86.0%)	0.661
	Gastrointestinal dysfunction		12 (75.0%)	28 (65.1%)	0.545
	Cardiovascular autonomic dysfunction		12 (75.0%)	15 (34.9%)	0.008
		Orthostatic hypotension	8 (50%)	9 (20.9%)	0.05
		POTS	8 (50%)	4 (9.3%)	0.002
		Palpitation	2 (12.5%)	3 (7.0%)	0.606
	Bladder dysfunction		7 (43.8%)	16 (37.2%)	0.647
	Anhidrosis		7 (43.8%)	9 (20.9%)	0.104
**Sensory symptom**			14 (87.5%)	42 (97.7%)	0.176
	Pain		13 (81.3%)	39 (90.7%)	0.375
	Sensory disturbance		12 (75.0%)	31 (72.1%)	1.000
**Motor symptom**			14 (87.5%)	40 (93.0%)	0.606
	Weakness		12 (75.0%)	35 (81.4%)	0.718
	Involuntary movement		5 (31.3%)	30 (69.8%)	0.007
	Ataxia		2 (12.5%)	7 (16.3%)	1.000

POTS, postural orthostatic tachycardia syndrome; A.I., antibody index.

The results of common laboratory tests revealed no significant differences in peripheral blood inflammatory markers, such as white blood cell count and C-reactive protein level, or in CSF inflammatory markers, such as total cell count and total protein level, between the anti-gAChR antibody-positive and -negative patient groups. No patient had low CSF pressure (< 60 mmH_2_O) on lumbar puncture examination. The IgG index, which measures the ratio of IgG and albumin levels in the CSF vs. serum and is an indicator of IgG production in the CNS, was also within normal limits in both patient groups (Table 3). Analysis of serum autoantibodies revealed that the presence of anti-nuclear antibodies was significantly more common in the anti-gAChR antibody-positive than -negative group (46.7 vs. 12.5%, *p* = 0.006).

**Table 3:** Laboratory data for FNSD/CD patients according to anti-gAChR antibody serostatus.

	**Anti gAChR antibody**		
	**Positive**	**Negative**	***P*** **value**
Serum WBC count (/μl)	5,800.0 ± 1,065.6	6,248.1 ± 1,645.9	0.317
Serum CRP (mg/dl)	0.101 ± 0.178	0.037 ± 0.447	0.147
CSF cell count (/μl)	1.25 ± 0.45	1.22 ± 0.47	0.826
CSF protein (mg/dl)	22.60 ± 6.45	24.40 ± 11.97	0.573
IgG index	0.490 ± 0.041	0.483 ± 0.046	0.597
Anti nuclear antibody	7/15 (46.7%)	5/40 (12.5%)	0.006
Anti Tg antibody	0/15 (0%)	3/40 (7.5%)	NA
anti TPO antibody	0/15 (0%)	3/41 (7.3%)	NA
Anti GAD antibody	0/7 (0%)	0/25 (0%)	NA
Anti SS-A antibody	1/12 (8.3%)	0/39 (0%)	NA
Anti SS-B antibody	0/13 (0%)	0/38 (0%)	NA

Tg, thyroglobulin; TPO, thyroid peroxidase; GAD, glutamic acid decarboxylase.

## 4. Discussion

Among our cohort of FNSD/CD patients, more than two-thirds (88.1%) presented with autonomic symptoms and, notably, more than a quarter (27.1%) were positive for serum anti-gAChR antibodies. Autonomic symptoms have been reported to be a precursor to FNSD/CD and have been positively associated with autism (10, 20). However, to our knowledge, there have been no formal investigations of autonomic dysfunction in patients with FNSD/CD, possibly because the DSM-5 diagnostic criteria for FNSD/CD require the presence of motor and sensory symptoms but not of autonomic symptoms. Although motor and sensory symptoms in FNSD/CD patients can be ruled in or out as consistent with neurological dysfunction by neurologists, it is more difficult to rule out a neurological basis for autonomic symptoms. In the present study, we found high rates of autonomic symptoms (gastrointestinal and cardiovascular dysfunction) in addition to motor and sensory symptoms, were the main symptoms of FNSD/CD, suggesting that evaluation of autonomic symptoms is important for both the diagnosis of FNSD/CD and for understanding its pathophysiology.

Anti-gAChR antibodies were initially identified in sera from patients with autonomic symptoms, leading to the discovery of autoimmune autonomic neuropathy, however, they are rarely detected in other disorders or in healthy individuals. McKeon et al. reported that serum anti-gAChR antibodies were present in 155 of 15,000 patients (1.03%) with suspected paraneoplastic autoimmune neurological syndrome but in only 1 of 173 healthy subjects (0.58%) (21). More recently, 0% of 2,628 patients with non-AAG neurological diseases were positive for antibodies to α3-nAChR (22). These reports suggest that anti-gAChR antibodies are usually undetectable or present at very low levels in the serum (16, 19, 23). Although these reports used radioimmunoprecipitation assay (RIA) as the measurement method, it has been proven that RIA and LIPS show the similar performance to detect anti-gAChR antibody (19). The high prevalence of anti-gAChR antibodies among our FNSD/CD patient cohort (27.1%) prompted us to hypothesize that anti-gAChR antibodies may be directly associated with autonomic symptoms in these patients. A previous study suggests that patients' symptoms may differ depending on the antibody subtype (24). Unfortunately, the small number of our cases did not allow us to examine this point in the present study. Interestingly, we detected no significant difference in the frequency of autonomic symptoms between patients who were positive or negative for serum anti-gAChR antibodies, suggesting that a direct relationship between the antibodies and autonomic symptoms is unlikely.

Anti-nuclear antibodies were significantly elevated in patients positive for anti-gAChR antibodies in the present study. Previous studies have identified antibodies against nAChR α4 and α7 subunits, VGKC channels, amphiphysin, and SS-B in anti-gAChR antibody-positive sera, suggesting the presence of a variety of humoral immune responses in these individuals (25–27). Interestingly, some patients with CNS symptoms, such as impaired consciousness and seizures, are positive for anti-gAChR antibodies, and several reports suggest that α7 and α4β2 nAChRs are involved in memory and pain (12, 16, 21, 24, 28–31). Notably, recent functional MRI studies have revealed that disturbances in the central autonomic network (CAN) can lead to psychiatric symptoms (32–34). These observations suggest that antibodies targeting nAChR subunits may affect not only the peripheral autonomic nervous system but also CNS function. The detection of anti-gAChR antibodies in patients with FNSD/CD with or without autonomic symptoms in the present study suggests that anti-gAChR antibodies do not simply affect peripheral autonomic function. However, whether anti-gAChR antibodies are a pathogenic factor or simply correlate with disease status remains unclear.

## 5. Conclusions

More than 80% of our FNSD/CD patient cohort had autonomic symptoms, and more than 25% of the cohort were positive for serum anti-gAChR antibodies, whether or not autonomic symptoms were present. To clarify the relationship between this psychiatric disorder and anti-gAChR antibodies, further investigation of clinical symptoms and the establishment of a measurement system for antibodies against the anti-nAChR subunit are needed.

## Data availability statement

The raw data supporting the conclusions of this article will be made available by the authors, without undue reservation.

## Ethics statement

The studies involving human participants were reviewed and approved by Ethics Review Board on Bioethics and Genetic Research Reference of Kagoshima University (Approval No. 492). Written informed consent to participate in this study was provided by the participants' legal guardian/next of kin.

## Author contributions

RN, EM, and HT conceived and designed the study. SNo, MD, YN, MA, YHir, YHig, YS, HA, and KH provided the laboratory data for patients at Kagoshima University Hospital. SNa provided the laboratory data for control subjects. DK, MT, RK, MY, and TH analyzed the data, take responsibility for the integrity of the data, and the accuracy of the data analysis. All authors critically revised the manuscript and approved of the final version.
